# Compositional analysis of lymphocytes and their relationship with health outcomes: findings from the health and retirement study

**DOI:** 10.1186/s12979-025-00505-z

**Published:** 2025-03-12

**Authors:** Lantian Xu, Chihua Li, Allison E. Aiello, Kenneth M. Langa, Jennifer B. Dowd, Rebecca C. Stebbins, Helen C. S. Meier, Ziman Jiang, Grace A. Noppert, Gen Li

**Affiliations:** 1 Department of Biostatistics, School of Public Health, University of Michigan, Ann Arbor, MI USA; 2 Institute of Chinese Medical Sciences, University of Macau, Macao, SAR China; 3 Institute for Social Research, University of Michigan, Ann Arbor, MI USA; 4 Department of Epidemiology, Columbia University Mailman School of Public Health, New York, NY USA; 5 Robert N. Butler Columbia Aging Center, Mailman School of Public Health, Columbia University, New York, NY USA; 6 Department of Internal Medicine, School of Medicine, University of Michigan, Ann Arbor, MI USA; 7 Leverhulme Centre for Demographic Science, University of Oxford, Oxford, UK; 8 Nuffield College, University of Oxford, Oxford, UK

## Abstract

**Background:**

Immunosenescence, the gradual deterioration of the immune system, is critical for aging-related diseases. However, the lack of detailed population-level immune data has limited our understanding, underscoring the need for innovative analytical approaches. The Health and Retirement Study (HRS) in the United States provides a unique opportunity to examine T and B lymphocyte subsets using compositional data analysis and dimension reduction techniques.

**Methods:**

We constructed a hierarchical tree structure to map relationships among T and B subset cells in HRS. Network analysis examined conditional dependence across 16 immune subset cells, while stepwise redundancy analysis (SRDA) identified a subset of pairwise logratio measures that capture main variance in immune composition. We conducted two sets of supervised learning analyses: first, linear penalized log-contrast models to examine the associations between subset cells and three health outcomes (chronic disease index, self-reported health, and frailty level); second, linear regressions to examine the associations between the top selected logratios and health outcomes.

**Findings:**

Our study included 6,250 participants from the HRS with a median age of 68. Network analysis showed some dependence among 16 immune subset cells, including associations between central memory CD4 + T cells and both other CD4 + T cells and other lymphocytes, as well as between central memory CD8 + T cells and other CD8 + T cells. SRDA identified nine key log-ratio measures, explaining over 90% of the variance in immune composition. Linear penalized log-contrast models showed that a lower proportion of naïve CD4 + T cells and higher proportions of other CD4 + and central memory CD8 + T cells were significantly associated with greater chronic disease burden, poorer self-reported health, and higher frailty levels. Linear regression models using log-ratios reinforced these patterns, showing that a higher ratio of other lymphocytes over naïve CD4 + T cells and terminally differentiated effector memory CD4 + T cells over other CD8 + T cells were associated with greater chronic disease burden, poorer self-reported health, and higher frailty levels. In contrast, a higher ratio of other lymphocytes over central memory CD4 + T cells was associated with better health outcomes.

**Interpretation:**

Our findings highlight the value of a systems-based approach and compositional analysis in understanding immunosenescence and its impact on health. The identified subset cells and logratio measures provide meaningful insights into immune aging and warrant further investigation to explore their long-term relationships with health outcomes.

**Supplementary Information:**

The online version contains supplementary material available at 10.1186/s12979-025-00505-z.

## Introduction

The immune system experiences significant changes with age and exposure to various infections and psychosocial stressors [1–3]. These changes, collectively known as immunosenescence, include low-level chronic systemic inflammation, a reduction in naïve T cells, an accumulation of effector memory T cells with diminished function, and a reversal in the CD4+:CD8 + T cell ratio [4–6]. Understanding these immune shifts at a population level requires integrating perspectives from immunology, epidemiology, and biostatistics. An early attempt to identify a risk phenotype associated with immune system aging in population studies led to the development of the immune risk phenotype (IRP) [7–10]. This measure, which mainly consisted of CD8 + and CD4 + T cells and T cell proliferative response, was developed through the analysis of peripheral blood samples in cohorts of octogenarians and nonagenarians [7, 10]. However, IRP was not reproduced in larger studies and was also challenging to implement due to the multiple biomarkers required to determine this phenotype [11, 12]. 

In 2016, Aiello et al. further innovated these measures by examining ratios of CD4 and CD8 effector cells to naïve cells as indicators of the immune system’s shift toward an IRP [13]. They examined how the effector to naïve ratio, combined with CMV seropositivity, was associated with socioeconomic and stress-related exposures. Building on these findings, our group and others have applied these measures in the Health and Retirement Study (HRS) in the United States (U.S.), demonstrating the immune system is associated with social stress and environment.[4–6,14−17] Furthermore, these measures have also been used to examine the relationship between immunosenescence and various health outcomes in HRS, including type 2 diabetes mellitus, cardiovascular diseases, cancer, neurodegenerative disorders, frailty, and increased premature mortality [18–21]. Collectively, these studies provide valuable insights into the causes and consequences of immunosenescence.

Valid measures of immunosenescence are critically important to test hypotheses about its causes and consequences. To date, researchers have employed three main methods to construct lymphocyte-based measures for characterizing immunosenescence. These approaches include absolute counts of different subset cells (i.e. CD4 + and CD8 + T cells) [16, 18, 19, 22], percentages of specific subset cells relative to their parent population (i.e. the percentage of naïve CD4 + T cells over all CD4 + T cells) [4, 14, 18, 20], and ratios between various pairs of subset cells (i.e. CD4+: CD8 + T cell ratio, terminally differentiated effector memory: naïve CD4 + T cell ratio) [4–6, 14, 18, 20, 21]. While these measures have yielded important insights, they may not adequately capture the system-wide interactions among and within different lymphocyte subsets. Given the close interdependence between T and B cells, conceptualizing their relationships as part of an interconnected and dynamic network is crucial for advancing our understanding of immunosenescence [23–25]. 

T and B cells, key components of lymphocytes and the adaptive immune system, experience a decrease in the production of naïve cells and an increase in memory cells with aging and immunological challenges [26, 27]. It is important to note that the increase in memory cells is associated with CMV infection [22]. T cells respond to pathogens by directly attacking infected cells and producing cytokines, which mobilize other immune components [28]. B cells primarily produce specific antibodies that neutralize viruses and bacteria or mobilize other effector cells and molecules to destroy invading microorganisms [29]. In this process, T cells not only fend off infections but also support B cell maturation and antibody production, crucial for long-term immunity [28, 30]. The cooperative dynamics between these two subset cells are fundamental to a robust immune system. Therefore, the relationships among different subset cells are themselves important to understand as both predictors and endpoints related to health and disease [25]. 

It is critical to consider the biological role of different immune subset cells in immune defense and aging. Adequate numbers and diversity of naïve T and B cells are critical to the immune defense against new (e.g. West Nile virus, SARS-1, SARS-CoV-2) or reemerging mutating (e.g. influenza) infections, and both decline with aging, which happens earlier and more severely for naïve T cells, particularly CD8 cells [27, 31]. In this manner, reduced naïve T and B cells are expected to correlate with, and mechanistically contribute to, increased morbidity and mortality from new infections. Conversely, highly differentiated memory lymphocytes, again particularly T cells, can fuel dysregulated inflammation and tissue dysfunction by secreting one or more cytokines or chemokines that participate in these processes [32]. This would directly relate to the increased frequency and severity of age-related chronic diseases and geriatric syndromes. Recent studies have identified molecular markers or transcriptional signatures of such cells, including CD151 and granzyme K (GzmK), and their role is under intense investigation [24, 33–35]. 

In this study, we aim to characterize the interrelationships among available lymphocyte subset cells in HRS, identify which logratio measures capture main variance across all possible logratio measures, and examine how these subsets and their derived logratios relate to health outcomes. To achieve these aims, we employed a system-based approach and combined unsupervised and supervised learning methods. First, we built a tree structure to delineate relationships across T and B subset cells and construct corresponding compositional measures. Second, we applied unsupervised learning techniques to examine the inter-relationships among various subset cells and identified key logratio measures that explain the most significant variations. Finally, using supervised learning, we examined the associations between immune subset cells and logratios with multiple health outcomes. This integrated approach offers a comprehensive framework for analyzing immune system complexity in a large, population-based setting.

## Methods

### Study population and procedures

The Health and Retirement Study (HRS) is an ongoing nationally representative longitudinal survey of older adults in the U.S. It began in 1992 and included over 22,000 adults over the age of 50 years at baseline and interviewed every two years [17]. Data collection consisted of face-to-face baseline interviews and primarily telephone interviews for follow-up waves, until 2006, when half the sample (alternated at each subsequent wave) was randomly assigned face-to-face interviews to enhance physical and biological measures. In this study, we utilized data from the 2016 h survey that included venous blood samples collected from 9,932 participants during 2016-17.

All participants who completed an interview during the 2016 wave were asked to consent to a venous blood draw except for proxy respondents and nursing home residents. The request was made by their HRS interviewers at the end of the interview. Blood collection occurred in the participants’ homes, managed by Hooper Holmes Health & Wellness. The vast majority of the blood samples were collected within four weeks of the interview completion. Overall, 65% of eligible participants provided a blood sample. Blood collected in cell preparation tubes (CPTs) was shipped at room temperature to the Advanced Research and Diagnostics Laboratory at the University of Minnesota and processed within 48 h of collection. The samples were transported in Styrofoam-lined shipping containers with foam holders specifically designed for the CPTs. Additionally, 2–3 gel packs, maintained at room temperature, were placed outside the Styrofoam layer but within the cardboard container to minimize temperature fluctuations during transit. The CPTs were centrifuged to isolate peripheral blood mononuclear cells (PBMCs), which were then cryopreserved using established protocols and stored in liquid nitrogen freezers for future use [16, 36]. 

### Immunophenotyping and percentage measures

The immune subset cells were identified using minor modifications to the standardized protocol published by the Human Immunology Project [37]. Per these guidelines, large batch analysis of frozen and thawed PBMC was found to be superior in reproducibility, while reducing small-batch variability associated with fresh sample analysis. One vial of cryopreserved mononuclear cells containing ~ 4 million cells was thawed, and cells were incubated at 37 °C in Roswell Park Memorial Institute media for 1 h. The cells were centrifuged at 1 200 rpm for 10 min at room temperature. The cells were resuspended in 1× phosphate buffered saline and stained as outlined previously [38]. The cells were kept on ice until analysis. All flow cytometry measurements were performed on an LSRII flow cytometer or a Fortessa X20 instrument (BD Biosciences, San Diego, CA). The validity of the T cell distributions obtained from cryopreserved PBMCs using the procedures used in HRS has been demonstrated previously [38]. In addition, control samples from healthy volunteers collected and cryopreserved at the start of the study were analyzed at least twice per week using the study protocol to monitor laboratory shifts and drifts in the immunophenotyping assessments.

In this study, we focused on lymphocyte data and key components of the adaptive immune system: T and B cells. These cells were measured in the same panel based on previously published protocols [36, 38], and the immunophenotyping data were analyzed using OpenCyto and FlowAnnotator [39]. Table 1 presents 15 examined T and B subset cells, including the cell type, marker used to determine the cell, abbreviated name, and percentage measure. In HRS flow cytometry, T and B subset cells were initially measured as percentages, calculated as the proportion of events relative to their parent populations within the live lymphocyte gate after removing doublets. Each subset cell was represented as a percentage of its parent cell. For example, the percentage of T cells was calculated as ‘Total T cells/total lymphocyte*100’; the percentage of TCD4 + was calculated as ‘CD4 + T cells/total T cells*100’; the percentage of TCD4N was calculated as ‘CD4 + Naïve T cells/CD4 + T cells*100’.

**Table 1:** Available percentage measures of 15 major subsets of T and B cells

Number	Cell type	Marker	Abbreviated name	Percentage measure
1	T cells	CD3 + CD19-		Total T cells/total lymphocyte*100
2	CD4 + T cells	CD3 + CD19- CD8- CD4+	TCD4+	CD4 + T cells/total T cells*100
3	CD4 + T cells: Naïve	CD3 + CD19- CD8- CD4 + CD45RA + CCR7 + CD28+	TCD4N	CD4 + Naïve T cells/CD4 + T cells*100
4	CD4 + T cells: Central memory (CM)	CD3 + CD19- CD8- CD4 + CD45RA- CCR7 + CD28+	TCD4CM	CD4 + CM T cells/CD4 + T cells*100
5	CD4 + T cells: Effector memory (EM)	CD3 + CD19- CD8- CD4 + CD45RA- CCR7- CD28-	TCD4EM	CD4 + Tem T cells/CD4 + T cells*100
6	CD4 + T cells: Terminally differentiated effector memory (TDEM)	CD3 + CD19- CD8- CD4 + CD45RA + CCR7- CD28-	TCD4TDEM	CD4 + TemRA T cells/CD4 + T cells*100
7	CD8 + T cells	CD3 + CD19- CD8 + CD4	TCD8+	CD8 + T cells/total T cells*100
8	CD8 + T cells: Naïve	CD3 + CD19- CD8 + CD4- CD45RA + CCR7 + CD28+	TCD8N	CD8 + Naïve T cells/CD8 + T cells*100
9	CD8 + T cells: Central memory (CM)	CD3 + CD19- CD8 + CD4- CD45RA- CCR7 + CD28+	TCD8CM	CD8 + CM T cells/CD8 + T cells*100
10	CD8 + T cells: Effector Memory (EM)	CD3 + CD19- CD8 + CD4- CD45RA- CCR7- CD28-	TCD8EM	CD8 + Tem T cells/CD8 + T cells*100
11	CD8 + T cells: Terminally differentiated effector memory (TDEM)	CD3 + CD19- CD8 + CD4- CD45RA + CCR7- CD28-	TCD8TDEM	CD8 + TemRA T cells/CD8 + T cells*100
12	B cells	CD3- CD19+		Total B cells/total lymphocyte*100
13	Naive B cells	CD3- CD19 + IgD + CD27+	BN	Naive B cells/total B cells
14	IgD- Memory B cells	CD3- CD19 + IgD- CD27+	BMIgD-	IgD- memory B cells/total B cells
15	IgD + Memory B cells	CD3- CD19 + IgD + CD27-	BMIgD+	IgD + memory B cells/total B cells

While both percentage data and count data were provided in the HRS, we prioritized the use of percentage data for two reasons. First, percentages were the primary measurements obtained in the HRS, while counts were derived by multiplying these percentages by total lymphocyte counts from the complete blood count. Our analyses confirmed that results based on percentages were identical to those obtained using counts, as both reflect the same underlying immunophenotypes. Second, percentage data offer greater consistency for population-level analyses by standardizing measurements relative to parent populations. This reduces biases caused by inter-individual variability in total lymphocyte counts and facilitates meaningful comparisons across a large cohort.

### Tree structure and compositional measures

**Fig. 1:**
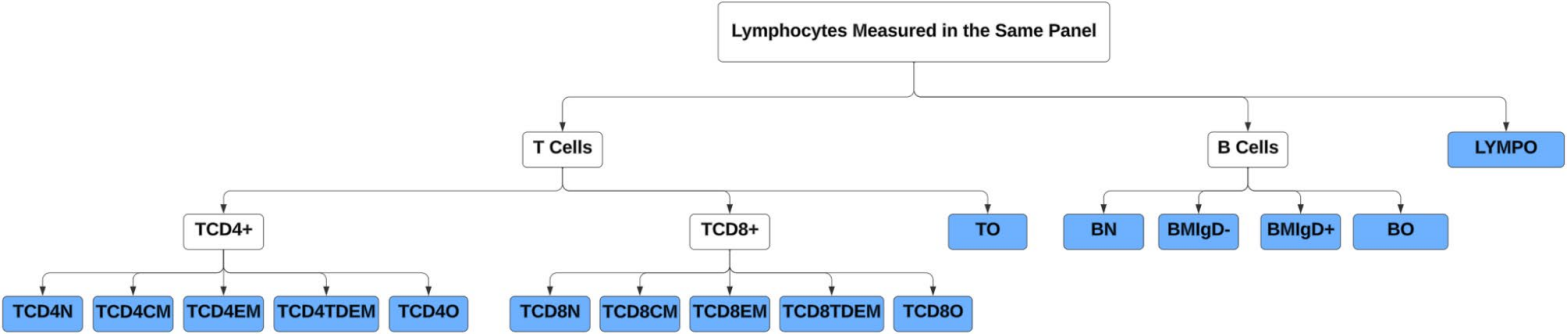
Hierarchical tree structure of T and B cell subsets. Footnote: The full names of the subset cells are summarized in Table 1. The subset cells highlighted with a blue background at the lowest level were included in our analysis

Based on the T and B subset cells in Table 1, we constructed a hierarchical tree structure to delineate the relationships among these cells across four levels of granularity (Fig. 1). In addition to the 15 subset cells provided by HRS, we introduced five additional subset cells because the sum of various subset cells at the lower level did not add up to 100% for their corresponding parent cells. For example, we introduced “other lymphocytes” because the combined percentage of T cells and B cells was less than 100%. The five newly defined five subsets included other lymphocytes (LYMPO), other B cells (BO), other T cells (TO), other CD4 + T cells (TCD4O), and other CD8 + T cells (TCD8O).

The percentage measures of subset cells in Table 1 used different denominators, making it difficult to examine and analyze them as a system. Based on the tree structure, we converted the percentage measures to compositional measures through several steps, allowing us to represent the proportion of each subset cell relative to the total lymphocyte count. First, we excluded 3,032 individuals with missing data on T and B cells and their subsets. We compared those with and without missing data and found they were comparable in main sociodemographic characteristics (Supplementary Table 1). Second, we excluded 39 participants because adding percentages of subset cells at the lower level exceeded 100%. Last, we generated the compositional measure for each subset cell by multiplying proportion measures at different levels so that the newly generated measure reflected the proportion of each subset cell in relation to the total lymphocyte count. The 16 subset cells at the most granular level (colored blue) in Fig. 1 were used for statistical analysis to provide the most detailed information and avoid redundancy.

### Health outcomes

We included three main health outcomes: a chronic disease index, self-reported health, and frailty level. The chronic disease index is the number of self-reported chronic conditions for each participant at the time of data collection in 2016 and ranges in value from 0 to 8. It is created by summing the number of affirmative responses to the following questions: Has a doctor ever told you that you have the following condition: (1) hypertension or high blood pressure; (2) diabetes or high blood sugar; (3) cancer or a malignant tumor of any kind except skin cancer; (4) chronic lung disease except asthma such as chronic bronchitis or emphysema; (5) heart attack, coronary heart disease, angina, congestive heart failure, or other heart problems; (6) stroke or transient ischemic attack; (7) emotional, nervous, or psychiatric problems; and (8) arthritis or rheumatism. The measure of self-reported health is represented by participants’ rate of their health status on a five-point scale ranging from 1 (excellent) to 5 (poor).

Frailty was measured using a deficit accumulation approach that included 44 variables reflecting multiple physiological systems in HRS [40–42]. These variables included self-reported chronic diseases, self-reported health, limitations in ADLs and IADLs, mobility restrictions, cognitive functioning, sensory impairments, somatic and depressive symptoms, BMI categories, and low physical activity. Each variable was scored from 0 (no deficit) to 1 (complete deficit), with intermediate values for ordinal or metric items. A continuous frailty index ranging from 0 to 1 was calculated as the total score divided by the maximum possible score. For interpretability, we categorized the frailty index into four ordered groups: robust (< 0.15), prefrail (0.15–0.24), mildly frail (0.25–0.34), and moderate-to-severely frail (≥ 0.35). These categories, assigned values from 1 to 4, allowed frailty to be examined as an ordered categorical outcome, consistent with the other two health measures. Across all three health outcomes, lower values indicate better health status. Correlations among these outcomes ranged from 0.37 to 0.60 (Supplementary Table 2), with the strongest correlation (*r* = 0.60) between the chronic disease index and frailty.

### Other covariates

Self-reported sociodemographic characteristics were collected in the 2016 core interview, including age, sex, race/ethnicity, and educational attainment. Sex was self-reported as either men or women. Race/ethnicity was categorized as non-Hispanic White, non-Hispanic Black, Hispanic, or Other Race. Educational attainment was categorized as below secondary education, lower secondary education, upper secondary education, and above upper secondary education. Cytomegalovirus (CMV) seropositivity was assessed using the Roche e411 immunoassay analyzer (Roche Diagnostics Corporation, Indianapolis, IN). The interassay coefficient of variation was 3.4% at a mean concentration of 1.2 COI (cutoff interval) and 2.9% at a mean concentration of 141.4 COI. Results were reported as nonreactive (< 1.0 COI) or reactive (≥ 1.0 COI). Since CMV is known to affect immune cell composition, especially effector T cells and memory T cells, we included CMV status (nonreactive or reactive) as a covariate to account for its possible influence on immune-health associations. A total number of 611 participants without information on these covariates were excluded. The final analytical sample included 6,250 participants, and the flow chart is shown in Supplementary Fig. 1.

### Statistical analysis

All analyses were conducted using R version 4.2. We examined correlations among the 16 subset cells at the most granular level in Fig. 1. We conducted both unsupervised and supervised learning analyses on lymphocyte compositional measures. The unsupervised learning focuses on network analysis of lymphocyte cell types with sparse inverse covariance estimation for ecological association inference (SPIEC-EASI) and dimension reduction with stepwise redundancy analysis (SRDA) [43, 44]. The supervised learning explores the association between health outcomes and lymphocyte cell types using a linear penalized log-contrast model. To facilitate the analysis, we substituted the smallest percentage of cell type in the dataset for 2883 participants with a subset cell value of 0%.

We used the SpiecEasi package to conduct a network analysis and examine the conditional dependence structure among the 16 subset cells. The method estimates an undirected, weighted network from compositional data using the covariance selection method (GLASSO) or neighborhood selection method (MB) [45]. In addition, we used the easyCODA package to identify a subset of pairwise logratios that can effectively capture the complete lymphocytes dataset [46]. Logratios are widely used when analyzing compositional data due to their subcompositional coherence property [45]. However, the extensive nature of the complete set of pairwise logratios presents a significant analytical challenge, necessitating a reduction in redundancy for practical application [44]. We analyzed 120 pairwise logratios constructed based on the 16 subset cells. In the first instance, we introduce all logratios and chose one of the logratios which would explain the highest percentage of the total variance. The second logratio that, independent from the chosen one, explained the largest percentage of variance was chosen from the remainder of the logratios, and the process iterated until 100% of the total variance was accounted for.

We conducted two sets of supervised learning analyses. First, we applied a linear penalized log-contrast model to examine the relationships between selected health outcomes and the 16 subset cells [47]. This model allows for both variable selection and estimation while accounting for the structure of compositional data. Variable selection was achieved by including a penalization term in the objective function. The tuning parameters were selected based on Bayesian information criterion (BIC) [48], and 95% confidence intervals were constructed using the bootstrap with 200 repetitions. Second, we used linear regression to assess the associations between selected health outcomes and selected logratio measures, which together capture 90% of the total variance in immune cell composition. For each health outcome, we fitted three models: (1) using either the 16 immune subset cells or the top logratio measures as predictors; (2) adjusting for age and gender; (3) further adjusting for race, education attainment, and CMV.

## Results

Participants included in our analysis had a median age of 68 years (IQR range: 62.0, 77.0), and 57.4% were women (Table 2). The majority of the participants were Non-Hispanic White (65.0%) and achieved an education level of high school or above (80.6%). The median chronic disease index was 2.0 with a higher score indicating more self-reported chronic conditions. The median self-reported health score was 3.0 (good) with a higher score indicating worse self-reported health. The median proportion for each type of 16 subset cells ranges from 0.07 to 21.1%. Details on distributions of 16 subset cells of lymphocytes included in the analysis are also summarized in Table 2. Their correlations with each other are presented in Supplementary Fig. 2.

**Table 2:** Demographic characteristics of the health and retirement study analytic sample

	Median/Count	IQR/Percentage
**Age (years)**	68	(62.0, 77.0)
**Gender**		
Men	2,660	42.6%
Women	3,590	57.4%
**Race**		
Non-Hispanic White	4,060	65.0%
Non-Hispanic Black	1,075	17.2%
Hispanic	915	14.6%
Other	200	3.2%
**Education**		
Below secondary	546	8.7%
Lower secondary	670	10.7%
Upper secondary	1,880	30.1%
Above upper secondary	3,154	50.5%
**CMV**		
Non-reactive	1,785	28.6%
Reactive	4,465	71.4%
**Chronic disease index**	2	(1.00, 3.00)
**Self-reported health**	3	(2.00, 4.00)
**Frailty level**	2	(1.00, 3.00)
**T cells**		
TCD4N (%)	19.8	(12.50, 28.50)
TCD4CM (%)	17.3	(13.50, 21.50)
TCD4EM (%)	0.07	(0.00, 0.38)
TCD4TDEM (%)	0.53	(0.18, 1.56)
TCD4O (%)	6.46	(4.39, 8.91)
TCD8N (%)	2.79	(1.72, 4.37)
TCD8CM (%)	1.09	(0.67, 1.74)
TCD8EM (%)	0.11	(0.03, 0.32)
TCD8TDEM (%)	6.28	(3.29, 11.30)
TCD8O (%)	2.69	(1.63, 4.14)
TO (%)	4.06	(2.74, 6.13)
**B cells**		
BN (%)	3.8	(2.27, 6.00)
BMIgD- (%)	0.53	(0.30, 0.91)
BMIgD+ (%)	0.58	(0.32, 1.01)
BO (%)	0.52	(0.31, 0.86)
**Other lymphocytes**		
LYMPO (%)	21.1	(14.50, 30.00)

**Fig. 2:**
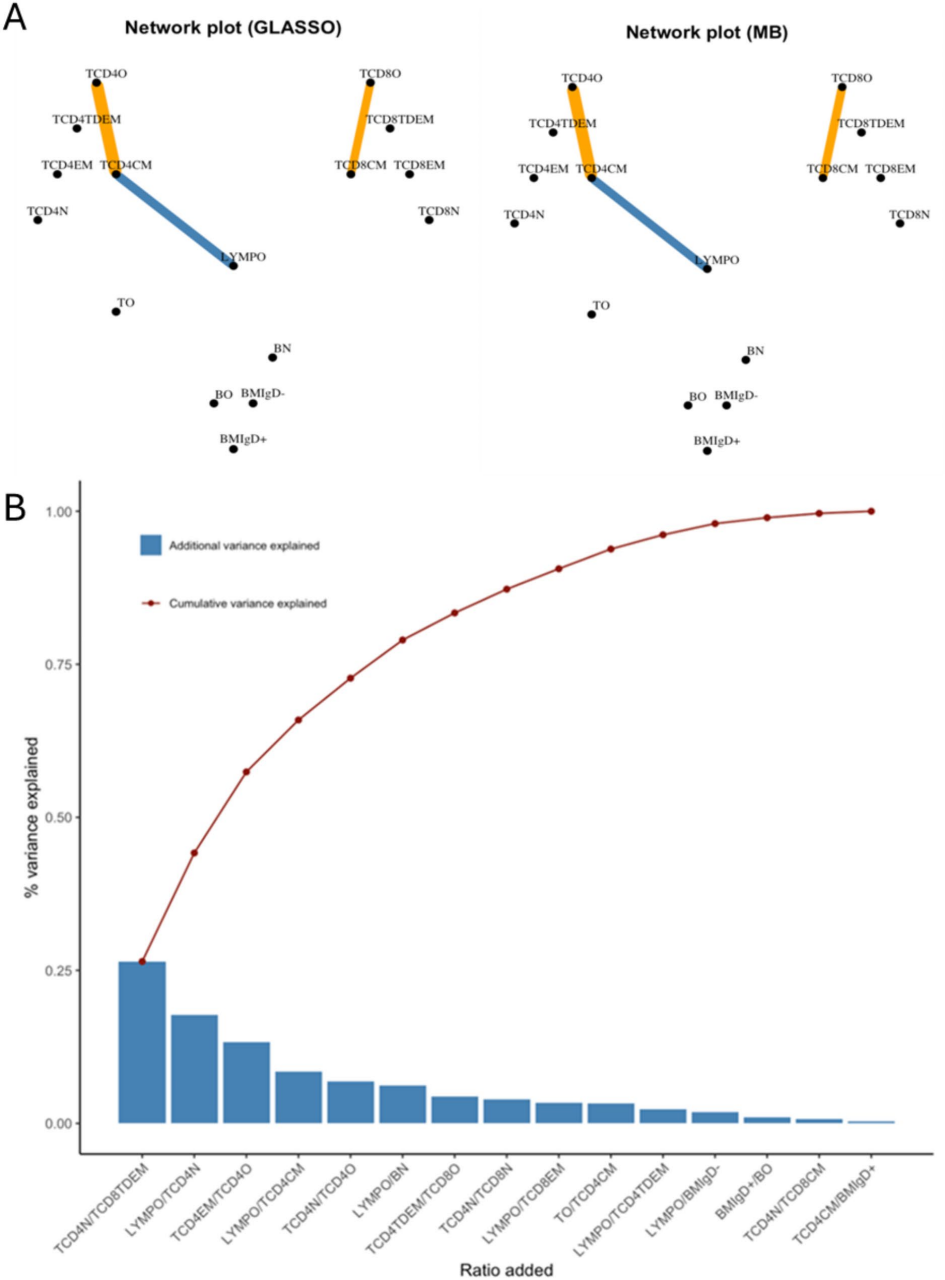
Results of unsupervised learning analyses using sparse inverse covariance estimation for ecological association inference (SPIEC-EASI) and stepwise redundancy analysis (SRDA). Footnote: Panel A shows pair-wise associations among subset cells based on SPIEC-EASI, with orange lines indicating positive associations and blue lines indicating negative correlations. Panel B shows additional and cumulative variances of by each logratio among the 15 linearly independent logratios based on SRDA

SPIEC-EASI showed pair-wise associations among subset cells when adjusting for all other cell types based on both GLASSO and MB methods (Fig. 2A). Both methods produced similar findings. Five subset cells and three pairs were associated with each other. After adjusting for all other cell types, TCD4CM was positively associated with TCD4O and negatively associated with LYMPO. TCD8CM was positively associated with TCD8O. Based on the 16 subset cells, a total number of 120 possible logratios were generated, having a total variance of 0.5767. Out of the 120 logratios, 15 of them were linearly independent, explaining 100% of the total logratio variance (Fig. 2B). SRDA showed that log(TCD4N/TCD8TDEM) explained 26.5% of the total variance. Following this, log(LYMPO/TCD4N) emerged as the next most significant contributor, explaining an additional 17.7% of the total variance. The first nine selected logratios explain 90.6% of the total variance, which can be used to effectively characterize the dataset (Supplementary Table 3).

Table 3 shows associations between the 16 subset cells and three health outcomes using linear penalized log-contrast models. For chronic disease index, TCD4N, TCD8O, and BMIgD + were negatively associated with the number of chronic diseases across the three models, while TCD4O and TCD8CM were positively associated with the number of chronic diseases. For self-reported health, only models 1 and 2 had non-zero coefficient estimates for the 16 subset cells. Across the two models, TCD4N was associated with better health while TCD4EM was associated with worse health. TCD4N and TCD4EM were also the two most important subset cells when predicting self-reported health in the solution path plot for Model 3 (Supplementary Fig. 3). For frailty level, TCD4N and TCD8O were negatively associated with severity across all three models, while TCD4O and TCD8CM were positively associated. This pattern of associations was very similar to the results observed for the chronic disease index.

**Table 3:** Associations between 16 cell subsets of lymphocytes and health outcomes of chronic disease index, self-reported health, and frailty level

Chronic disease index (range: 0 to 8)			
Predictors	Model 1	Model 2	Model 3
TCD4N	-0.143 (-0.22, -0.11)	-0.185 (-0.31, -0.16)	-0.17 (-0.24, -0.11)
TCD4CM	0.078 (0, 0.2)	0.133 (0.07, 0.24)	0.095 (0.02, 0.2)
TCD4EM	0.013 (0, 0.02)	0.002 (-0.01, 0.01)	-0.003 (-0.02, 0)
TCD4TDEM	0 (-0.04, 0.02)	0 (-0.04, 0.02)	-0.012 (-0.05, 0)
TCD4O	0.106 (0.07, 0.18)	0.088 (0.05, 0.17)	0.103 (0.06, 0.17)
TCD8N	-0.064 (-0.12, -0.02)	0 (0, 0.07)	0 (-0.04, 0.02)
TCD8CM	0.142 (0.09, 0.22)	0.068 (0.02, 0.15)	0.103 (0.04, 0.18)
TCD8EM	0.012 (0, 0.03)	0.01 (0, 0.02)	0.01 (0, 0.03)
TCD8TDEM	0 (-0.03, 0.06)	0 (-0.02, 0.05)	0 (0, 0.05)
TCD8O	-0.221 (-0.32, -0.18)	-0.133 (-0.25, -0.1)	-0.145 (-0.23, -0.09)
TO	0 (0, 0.07)	0 (0, 0.07)	0 (0, 0.06)
BN	0 (-0.1, 0)	0.01 (0, 0.07)	0.038 (0, 0.09)
BMIgD-	-0.025 (-0.14, 0)	0 (-0.09, 0)	0 (-0.07, 0)
BMIgD+	-0.091 (-0.13, -0.03)	-0.053 (-0.12, -0.02)	-0.089 (-0.14, -0.04)
BO	0.082 (0.04, 0.21)	0.059 (0.02, 0.13)	0.032 (0, 0.1)
LYMPO	0.112 (0.06, 0.17)	0 (0, 0.08)	0.038 (0, 0.1)
Age	-	0.037 (0.03, 0.04)	0.035 (0.03, 0.04)
Gender: Female	-	0.145 (0.08, 0.23)	0.126 (0.06, 0.2)
Race: Black	-	-	0.254 (0.14, 0.36)
Race: Hispanic	-	-	-0.175 (-0.31, -0.06)
Race: Other	-	-	0.05 (-0.13, 0.31)
Education: Lower secondary	-	-	0.317 (0.14, 0.53)
Education: Upper secondary	-	-	-0.079 (-0.23, 0.12)
Education: Above upper secondary	-	-	-0.246 (-0.4, -0.05)
CMV: Reactive	-	-	0.078 (-0.01, 0.18)
Intercept	2.442 (2.17, 2.68)	-0.071 (-0.46, 0.23)	-0.059 (-0.55, 0.37)
**Self-reported health (range: 1 to 5)**			
**Predictors**	**Model 1**	**Model 2**	**Model 3**
TCD4N	-0.142 (-0.17, -0.06)	-0.152 (-0.18, -0.06)	0 (-0.1, 0)
TCD4CM	0.035 (0, 0.11)	0.04 (0, 0.1)	0 (0, 0.05)
TCD4EM	0.016 (0.01, 0.02)	0.015 (0.01, 0.02)	0 (0, 0.01)
TCD4TDEM	0.012 (0, 0.03)	0.01 (0, 0.03)	0 (-0.02, 0.01)
TCD4O	0.075 (0, 0.11)	0.075 (0.02, 0.1)	0 (0, 0.09)
TCD8N	0.024 (0, 0.05)	0.027 (0, 0.05)	0 (-0.01, 0)
TCD8CM	0.044 (0, 0.08)	0.044 (0, 0.08)	0 (0, 0.1)
TCD8EM	0 (-0.01, 0.01)	0 (-0.01, 0.01)	0 (-0.01, 0.01)
TCD8TDEM	0 (-0.01, 0.03)	0 (-0.01, 0.03)	0 (0, 0.04)
TCD8O	-0.101 (-0.14, 0)	-0.097 (-0.14, 0)	0 (-0.1, 0)
TO	0 (-0.04, 0)	0 (-0.04, 0.01)	0 (-0.03, 0)
BN	-0.016 (-0.06, 0)	-0.016 (-0.06, 0)	0 (-0.06, 0)
BMIgD-	0 (-0.02, 0.03)	0 (-0.02, 0.02)	0 (-0.04, 0)
BMIgD+	-0.033 (-0.07, 0)	-0.035 (-0.07, 0)	0 (-0.07, 0)
BO	0.036 (0, 0.07)	0.035 (0, 0.07)	0 (0, 0.05)
LYMPO	0.048 (0, 0.08)	0.053 (0, 0.08)	0 (0, 0.09)
Age	-	0.002 (0, 0)	0.005 (0, 0.01)
Gender: Female	-	0.073 (0.01, 0.13)	0.02 (-0.02, 0.1)
Race: Black	-	-	0.3 (0.21, 0.38)
Race: Hispanic	-	-	0.311 (0.22, 0.38)
Race: Other	-	-	0.331 (0.21, 0.48)
Education: Lower secondary	-	-	-0.068 (-0.18, 0.05)
Education: Upper secondary	-	-	-0.35 (-0.45, -0.23)
Education: Above upper secondary	-	-	-0.604 (-0.69, -0.49)
CMV: Reactive	-	-	0.047 (-0.05, 0.08)
Intercept	3.098 (2.93, 3.23)	2.929 (2.66, 3.13)	2.806 (2.58, 3.16)
**Frailty level (range: 1 to 4)**			
**Predictors**	**Model 1**	**Model 2**	**Model 3**
TCD4N	-0.126 (-0.17, -0.08)	-0.175 (-0.24, -0.14)	-0.115 (-0.14, -0.06)
TCD4CM	0.044 (0, 0.13)	0.081 (0.01, 0.17)	0.035 (0, 0.1)
TCD4EM	0.021 (0.01, 0.03)	0.014 (0, 0.02)	0.003 (-0.01, 0.01)
TCD4TDEM	0.013 (0, 0.04)	0.011 (0, 0.03)	-0.011 (-0.03, 0.01)
TCD4O	0.117 (0.07, 0.16)	0.104 (0.06, 0.15)	0.113 (0.06, 0.14)
TCD8N	-0.019 (-0.05, 0)	0.02 (0, 0.06)	0 (-0.02, 0)
TCD8CM	0.103 (0.05, 0.14)	0.058 (0.02, 0.1)	0.102 (0.04, 0.13)
TCD8EM	0 (-0.01, 0.01)	0 (-0.01, 0.01)	0.001 (-0.01, 0.01)
TCD8TDEM	0 (-0.03, 0.03)	0 (-0.03, 0.03)	0 (0, 0.03)
TCD8O	-0.211 (-0.26, -0.15)	-0.141 (-0.2, -0.1)	-0.144 (-0.17, -0.07)
TO	0 (-0.05, 0.02)	0 (-0.02, 0.03)	0 (-0.01, 0.02)
BN	-0.042 (-0.1, 0)	0 (-0.05, 0.02)	0 (-0.03, 0.02)
BMIgD-	0 (-0.06, 0.02)	0 (0, 0.03)	0 (-0.02, 0.02)
BMIgD+	-0.051 (-0.08, -0.01)	-0.035 (-0.08, 0)	-0.06 (-0.09, -0.02)
BO	0.09 (0.05, 0.16)	0.063 (0.03, 0.1)	0.041 (0, 0.07)
LYMPO	0.058 (0.01, 0.1)	0 (0, 0.05)	0.036 (0, 0.06)
Age	-	0.025 (0.02, 0.03)	0.023 (0.02, 0.03)
Gender: Female	-	0.253 (0.19, 0.3)	0.23 (0.17, 0.28)
Race: Black	-	-	0.3 (0.23, 0.38)
Race: Hispanic	-	-	0.07 (-0.02, 0.17)
Race: Other	-	-	0.117 (-0.02, 0.27)
Education: Lower secondary	-	-	0.076 (-0.05, 0.19)
Education: Upper secondary	-	-	-0.264 (-0.38, -0.16)
Education: Above upper secondary	-	-	-0.552 (-0.66, -0.44)
CMV: Reactive	-	-	0.089 (0.02, 0.14)
Intercept	2.587 (2.41, 2.74)	0.773 (0.51, 0.96)	0.867 (0.57, 1.11)

Table 4 shows the associations between the selected nine logratios, which explain over 90% of the total variance across all possible logratios, and the three health outcomes using linear regression models. For the chronic disease index, LYMPO/TCD4N and TCD4TDEM/TCD8O were positively associated with a higher number of chronic conditions across models, while LYMPO/TCD4CM was negatively associated. For self-reported health, higher LYMPO/TCD4N was positively associated with worse health across models. LYMPO/TCD4CM and TCD4N/TCD4O were negatively associated with better health in models 1 and 2, but these associations weakened in model 3 after adjusting for additional covariates. For frailty level, LYMPO/TCD4N and TCD4TDEM/TCD8O were positively associated with higher frailty levels across models, while LYMPO/TCD4CM and TCD4N/TCD4O were negatively associated, mirroring the patterns observed for chronic disease index. Additionally, comparing models using the selected nine logratios versus all 120 pairwise logratios, the adjusted R² values were similar, indicating that the reduced model captures most of the explanatory power while offering a more interpretable representation of immune cell composition.

**Table 4:** Associations between 9 selected logratios and health outcomes of chronic disease index, self-reported health, and frailty level

Chronic disease index (range: 0 to 8)			
Predictors	Model 1	Model 2	Model 3
TCD4N/TCD8TDEM	0.038 (-0.01, 0.09)	0.043 (-0.01, 0.09)	0.03 (-0.02, 0.08)
LYMPO/TCD4N	0.322 (0.24, 0.4)	0.28 (0.2, 0.36)	0.261 (0.18, 0.34)
TCD4EM/TCD4O	0.005 (-0.01, 0.02)	-0.005 (-0.02, 0.01)	-0.009 (-0.02, 0)
LYMPO/TCD4CM	-0.206 (-0.28, -0.13)	-0.218 (-0.29, -0.15)	-0.189 (-0.26, -0.12)
TCD4N/TCD4O	-0.019 (-0.07, 0.03)	-0.03 (-0.08, 0.02)	-0.024 (-0.07, 0.03)
LYMPO/BN	0.021 (-0.02, 0.07)	-0.039 (-0.08, 0)	-0.033 (-0.08, 0.01)
TCD4TDEM/TCD8O	0.057 (0.03, 0.08)	0.05 (0.03, 0.07)	0.03 (0.01, 0.05)
TCD4N/TCD8N	0.114 (0.07, 0.16)	-0.017 (-0.07, 0.03)	0.021 (-0.03, 0.07)
LYMPO/TCD8EM	-0.023 (-0.04, -0.01)	-0.018 (-0.03, 0)	-0.018 (-0.03, 0)
Age	-	0.04 (0.04, 0.04)	0.037 (0.03, 0.04)
Gender: Female	-	0.147 (0.07, 0.22)	0.133 (0.06, 0.21)
Race: Black	-	-	0.232 (0.12, 0.34)
Race: Hispanic	-	-	-0.187 (-0.31, -0.06)
Race: Other	-	-	0.052 (-0.15, 0.26)
Education: Lower secondary	-	-	0.317 (0.15, 0.49)
Education: Upper secondary	-	-	-0.072 (-0.22, 0.08)
Education: Above upper secondary	-	-	-0.241 (-0.39, -0.09)
CMV: Reactive	-	-	0.046 (-0.06, 0.15)
Intercept	2.421 (2.27, 2.57)	-0.183 (-0.5, 0.13)	-0.055 (-0.44, 0.33)
**Self-reported health (range: 1 to 5)**			
**Predictors**	**Model 1**	**Model 2**	**Model 3**
TCD4N/TCD8TDEM	0.021 (-0.01, 0.06)	0.02 (-0.02, 0.06)	-0.001 (-0.04, 0.03)
LYMPO/TCD4N	0.112 (0.06, 0.17)	0.115 (0.06, 0.17)	0.061 (0.01, 0.12)
TCD4EM/TCD4O	0.013 (0, 0.02)	0.012 (0, 0.02)	0.002 (-0.01, 0.01)
LYMPO/TCD4CM	-0.074 (-0.12, -0.02)	-0.077 (-0.13, -0.03)	-0.042 (-0.09, 0.01)
TCD4N/TCD4O	-0.051 (-0.09, -0.02)	-0.052 (-0.09, -0.02)	-0.034 (-0.07, 0)
LYMPO/BN	0.02 (-0.01, 0.05)	0.019 (-0.01, 0.05)	0.041 (0.01, 0.07)
TCD4TDEM/TCD8O	0.04 (0.02, 0.06)	0.036 (0.02, 0.05)	0.015 (0, 0.03)
TCD4N/TCD8N	-0.022 (-0.05, 0.01)	-0.027 (-0.06, 0.01)	0.002 (-0.03, 0.04)
LYMPO/TCD8EM	-0.004 (-0.02, 0.01)	-0.004 (-0.02, 0.01)	-0.004 (-0.02, 0.01)
Age	-	0.003 (0, 0.01)	0.004 (0, 0.01)
Gender: Female	-	0.075 (0.02, 0.13)	0.057 (0, 0.11)
Race: Black	-	-	0.278 (0.2, 0.35)
Race: Hispanic	-	-	0.301 (0.22, 0.38)
Race: Other	-	-	0.334 (0.19, 0.48)
Education: Lower secondary	-	-	-0.063 (-0.18, 0.05)
Education: Upper secondary	-	-	-0.336 (-0.44, -0.23)
Education: Above upper secondary	-	-	-0.584 (-0.69, -0.48)
CMV: Reactive	-	-	-0.004 (-0.08, 0.07)
Intercept	3.103 (3, 3.21)	2.929 (2.66, 3.13)	2.936 (2.67, 3.2)
**Frailty level (range: 1 to 4)**			
**Predictors**	**Model 1**	**Model 2**	**Model 3**
TCD4N/TCD8TDEM	0.041 (0, 0.08)	0.041 (0, 0.08)	0.02 (-0.02, 0.06)
LYMPO/TCD4N	0.185 (0.13, 0.24)	0.172 (0.11, 0.23)	0.129 (0.07, 0.19)
TCD4EM/TCD4O	0.016 (0.01, 0.02)	0.009 (0, 0.02)	-0.001 (-0.01, 0.01)
LYMPO/TCD4CM	-0.132 (-0.19, -0.08)	-0.145 (-0.2, -0.09)	-0.106 (-0.16, -0.06)
TCD4N/TCD4O	-0.056 (-0.09, -0.02)	-0.064 (-0.1, -0.03)	-0.046 (-0.08, -0.01)
LYMPO/BN	0.021 (-0.01, 0.05)	-0.01 (-0.04, 0.02)	0.007 (-0.02, 0.04)
TCD4TDEM/TCD8O	0.068 (0.05, 0.08)	0.055 (0.04, 0.07)	0.028 (0.01, 0.05)
TCD4N/TCD8N	0.048 (0.02, 0.08)	-0.029 (-0.06, 0.01)	0.012 (-0.02, 0.05)
LYMPO/TCD8EM	-0.01 (-0.02, 0)	-0.007 (-0.02, 0)	-0.007 (-0.02, 0)
Age	-	0.026 (0.02, 0.03)	0.025 (0.02, 0.03)
Gender: Female	-	0.258 (0.2, 0.31)	0.236 (0.18, 0.29)
Race: Black	-	-	0.298 (0.22, 0.37)
Race: Hispanic	-	-	0.068 (-0.02, 0.15)
Race: Other	-	-	0.121 (-0.02, 0.27)
Education: Lower secondary	-	-	0.076 (-0.04, 0.2)
Education: Upper secondary	-	-	-0.259 (-0.37, -0.15)
Education: Above upper secondary	-	-	-0.554 (-0.66, -0.45)
CMV: Reactive	-	-	0.051 (-0.02, 0.12)
Intercept	2.494 (2.38, 2.6)	0.645 (0.42, 0.87)	0.801 (0.53, 1.07)

## Discussion

In this study, we examined relationships among subsets of T and B cells and their relationship with health outcomes using the HRS venous blood data. We had three main findings: first, we found three pairs of subset cells associated with each other when adjusting for all other subset cells, including TCD4CM and TCD4O, TCD4CM and LYMPO, and TCD8CM and TCD8O. Second, we identified nine logratios explaining over 90% of the total variance, indicating they well represent the dataset of T and B cells. Third, we found multiple subset cells associated with health outcomes of chronic disease index and self-reported health, including TCD4N, TCD4EM, TCD4O, TCD8CM, TCD8O, and BMIgD+. These findings together suggested a relatively low correlation across subsets of T and B cells and the importance of considering multiple subset cells as a complex system when examining the relationship between immunosenescence and health outcomes.

We constructed a tree structure of available T and B subset cells. The newly defined five subsets of ‘other cells’ showed the presence of additional subsets not fully captured through immunophenotyping conducted by HRS. Network analysis using SPIEC-EASI revealed much sparser interrelationships among subset cells than expected, suggesting that the majority of subset cells were conditionally independent. The sparse interrelationships among T and B subset cells have not been extensively explored or reported in previous studies using population health data. Multiple ‘other cells’ were associated with either TCD4M or TCD8O, indicating the existence of important, unmeasured cell types of interest. It is interesting to observe that TCD4CM was positively associated with TCD4O, and TCD8CM was positively associated with TCD8O. Since the younger (naïve cells) and older stage cells (effector memory cells and terminally differentiated effector memory cells) have been identified, these ‘other cells’ are more likely to be in the intermediate stage.

Contrary to our expectations, our network analysis did not reveal a negative association between naïve and terminally differentiated effector memory cells in both TCD4 + and TCD8 + subsets. This absence of a negative correlation can be attributed to the methodological approach of network analysis, which examines correlations between each pair of subset cells while adjusting for all other subsets. Interestingly, upon analyzing pairwise correlations across 16 subset cells, we indeed observed negative correlations between naïve and terminally differentiated effector memory cells for both TCD4 + and TCD8+.

Using SRDA, we further found that nine logratios explain over 90% of the total variance. The reduction from 15 independent logratios to nine suggests a relatively low correlation among them. This was also consistent with findings based on the network analysis that most subset cells were independent when adjusting for all other subset cells. A closer examination of these nine logratios revealed that some subset cells, including TCD4N and LYMPHO, appeared multiple times, highlighting critical subset cells. TCD4N is pivotal for maintaining a responsive and adaptable immune system [27, 31]. A decline in the number of TCD4N or functional impairment of these cells with age is a key factor in immunosenescence and affects the body’s ability to respond to new infections, generate effective memory responses, and maintain a balanced immune regulation [49]. This ultimately contributes to increased susceptibility to infections, reduced vaccine efficacy, and a higher risk of autoimmunity and inflammation-related diseases in the elderly [31, 50]. 

These logratio measures were identified from the perspective of compositional data analysis, making it challenging to interpret their biological meanings, especially since they include unmeasured cell types. However, some measures are newly identified and worth further exploration. Specifically, the log(TCD4N/TCD8TDEM) accounted for 26.5% of the total variance, a higher value of which may suggest less advanced immunosenescence. In future HRS studies, researchers can examine how these measures relate to age-related health outcomes over time, such as cognitive health, kidney function, and geriatric syndromes.

In supervised learning, we identified several subset cells associated with chronic disease index, self-reported health, and frailty level on a cross-sectional basis. TCD4N was negatively associated with both a higher number of chronic diseases and poorer health, and TCD4CM and TCD4O were positively associated with these outcomes. This is consistent with findings from other studies, suggesting these subset cells of TCD4 + may be useful biomarkers to identify individuals at higher risk of chronic diseases [18, 51–54]. Subset cells of TCD8 + were found to be associated with chronic disease index but not self-reported health. The direction of associations followed our expectation that TCD8N was negatively associated with a higher number of chronic diseases and TCD8CM was positively associated with a higher number of chronic diseases.

Furthermore, our analysis showed that TCD4N and TCD8O were negatively associated with frailty levels across all models, whereas TCD4O and TCD8CM were positively associated. This pattern mirrors closely the associations observed with the chronic disease index, underscoring the robustness of our findings across different health outcomes. The consistent relationships between these subset cells and multiple health measures suggest that immunosenescence markers are closely related to various aspects of health in older adults. These results highlight the potential of specific T subset cells as indicators of health status, reinforcing the importance of immune profiling in aging populations.

The analysis based on logratios reinforced these associations observed in the subset cell analysis, demonstrating that specific immune composition patterns are related to multiple health outcomes. For example, a higher LYMPO/TCD4N ratio was associated with greater chronic disease burden, poorer self-reported health, and higher frailty, suggesting that a higher proportion of other lymphocytes relative to naïve TCD4 + cells reflects an aging immune profile linked to poorer health. Conversely, LYMPO/TCD4CM was negatively associated with health outcomes, indicating that a higher ratio of other lymphocytes over TCD4 + central memory cells may be protective against immune-related health decline. Both results based on subset cells and logratios showed that a decline in naïve TCD4 + cells relative to other lymphocytes may indicate an aging immune profile contributing to poor health. Again, we observed findings of highly similar patterns based on both chronic disease index and frailty. Finally, by comparing R [2] values from models with different sets of predictors, we found that the selected nine logratios explained a similar amount of variation in the three health outcomes as the full set of 120 pairwise logratios. This confirms that the selected logratios effectively capture key aspects of immune composition while simplifying the model.

There were several major strengths of this study. First, it used a large U.S. population-representative sample (> 6,000 individuals) that implemented standardized immunophenotyping, providing a snapshot of T and B cells among older adults in the U.S. Second, the study expanded beyond individual subset cells or ratio measures to take the relationships among subset cells into consideration. It constructed a tree structure of available T and B subset cells and analyzed them as an interconnected system. Third, this study was pioneering in applying both unsupervised and supervised learning techniques to the complex cellular data of the Health and Retirement Study (HRS). These methods complemented each other and enabled a more nuanced understanding of the cellular landscape and its implications for health and disease.

The compositional and hierarchical tree framework developed in this study provides a methodological foundation for analyzing immune phenotypes across cohorts with similar immunophenotyping data. By treating lymphocyte subset cells as interdependent components of the immune system, this approach enables a scalable framework that can facilitate cross-cohort comparisons and enhance the robustness of findings. Furthermore, the key measures of T and B subset cells identified in this study, such as naïve CD4 + T cells and central memory T cells, may serve as potential biomarkers of immunosenescence and its association with health outcomes. Future research can leverage these methods in diverse demographic, geographic, and longitudinal contexts to validate and expand upon our findings. In resource-constrained environments, our study highlights specific immune biomarkers that may be prioritized for a better understanding of the biological processes of aging. This approach provides a foundation for advancing our understanding of immune system dynamics and their critical role in healthy aging and disease.

Our study also had several limitations. First, the cross-sectional design of our study limits the ability to assess causality or examine temporal trends in immunosenescence and its associations with health outcomes. Having data from only one-time point in the HRS provides only a snapshot in time of immune cell distributions, which cannot fully capture the complex and dynamic nature of immune system aging. To address this limitation, we recommend that future waves of HRS prioritize the collection of longitudinal immune data. Such an approach would enable the investigation of within-individual changes in immune profiles over time and their relationship with the onset or progression of age-related diseases. Linking multiple time points of immunophenotyping data with comprehensive health records would offer a robust framework for identifying predictive biomarkers and understanding the long-term impacts of immunosenescence on health outcomes. In future work, we plan to extend this research by examining how changes in these immune subset cells over time relate to the development and progression of various health outcomes. Second, while our study offers valuable insights into the associations between lymphocyte subsets and health outcomes, it is limited by the scope of immune measurements available in the HRS. For example, the HRS dataset does not include certain immune biomarkers such as activation and exhaustion markers, cytokine profiles, and antibody titers, which are crucial for a comprehensive understanding of immunosenescence. Future research should aim to incorporate these measures to provide a more detailed picture of immune aging and its health implications.

Third, our cross-sectional analysis of HRS highlights associations between immune cells and health outcomes but does not establish causality between them. Future research involving longitudinal data and more advanced causal methodologies will be essential for studying the underlying mechanisms of immunosenescence and its impact on aging. Such designs would capture dynamic changes in immune profiles, clarify temporal relationships, identify causal pathways, and examine how modifiable factors (e.g., lifestyle and therapeutic interventions) influence immune aging and associated health risks. Fourth, a high proportion of participants (30.5%) lacked complete cell data. However, we found no meaningful sociodemographic differences between participants with and without complete data, suggesting that the missing data may not significantly bias the study’s overall findings. Last, no specific information on immunosuppressive treatments was available, which prevents us from controlling their potential effects on both immune function and health outcomes. In the future, it is possible to link HRS data to Medicare claims to examine if information on immunosuppressive treatments can be retrieved.

In conclusion, we applied a system-based approach that combined unsupervised and supervised learning methods to analyze lymphocytes in a large U.S. population-based study. We found that the majority of subset cells are conditionally independent, and nine logratios effectively represent lymphocyte data. This indicates their potential as useful indicators for developing measures of immunosenescence. Furthermore, we identified multiple subset cells and logratio measures associated with different health outcomes, suggesting their systemic role in influencing health outcomes. This work is particularly relevant due to the increasing interest in targeting the peripheral immune system for aging-related diseases.

## Electronic supplementary material

Below is the link to the electronic supplementary material.

Supplementary Material 1

## Data Availability

The HRS data are publicly available at https://hrs.isr.umich.edu/about. All code to generate findings reported in this study are available from the GitHub repository (https://github.com/lxttw/Compositional-DA-HRS).
