# Supplementary material for: Compositional analysis of lymphocytes and their relationship with health outcomes: findings from the health and retirement study
Source: Immun Ageing. 2025 Mar 12;22:12. doi: 10.1186/s12979-025-00505-z (PMC11899731; doi:10.1186/s12979-025-00505-z)
Supplement: Supplementary file 1 — Supplementary Material 1 [file 12979_2025_505_MOESM1_ESM.docx]

**Supplementary Figure 1. Flow chart of analytical sample**


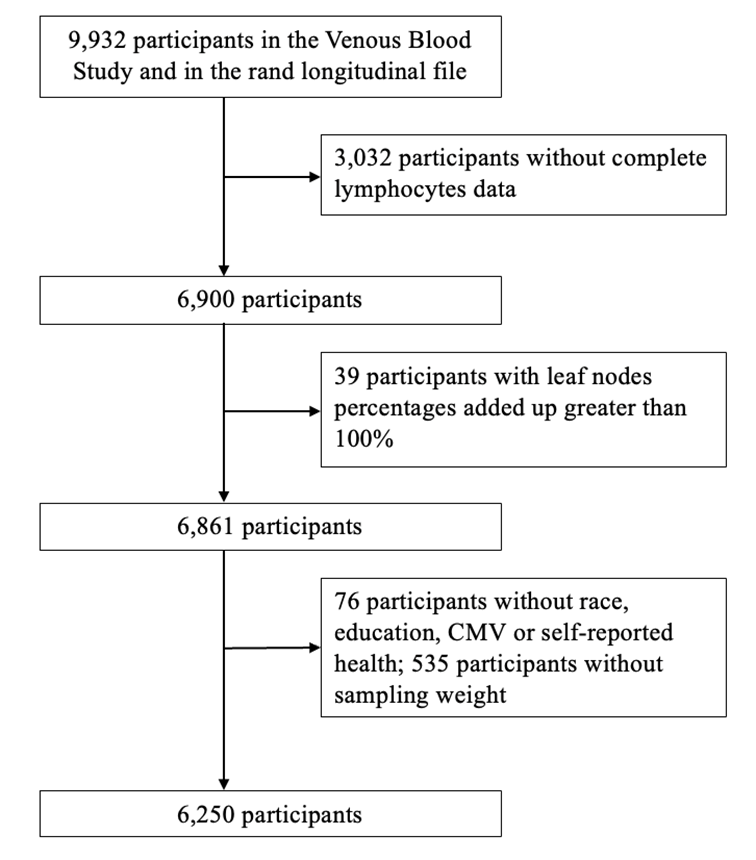


**Supplementary Figure 2. Correlation heatmap of 16 subset cells of lymphocytes at the most granular level**

**
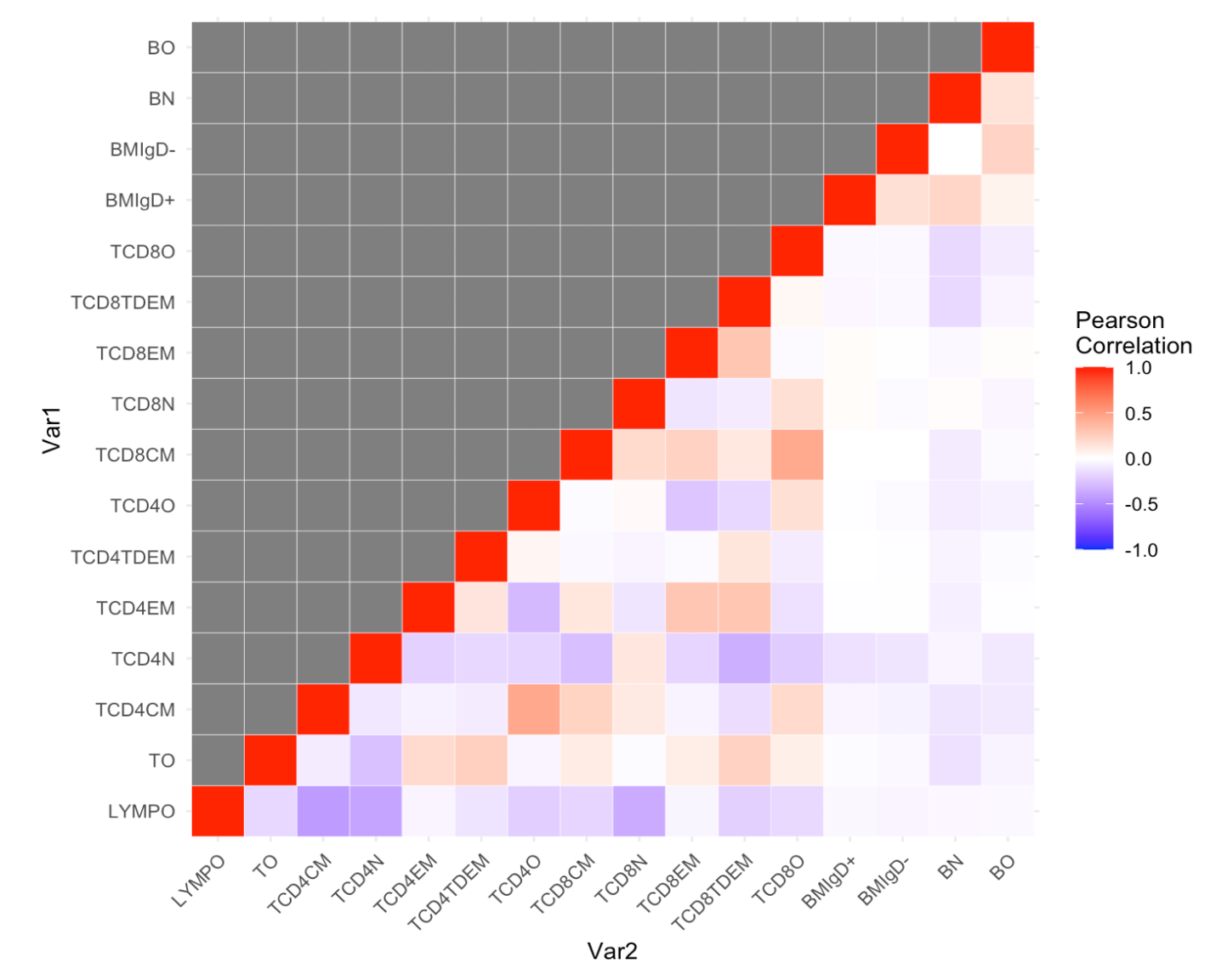
**

**Supplementary Figure 3. Solution path for the linear penalized log-contrast model with self-reported health, adjusted for age, gender, race, education attainment, and CMV**

**
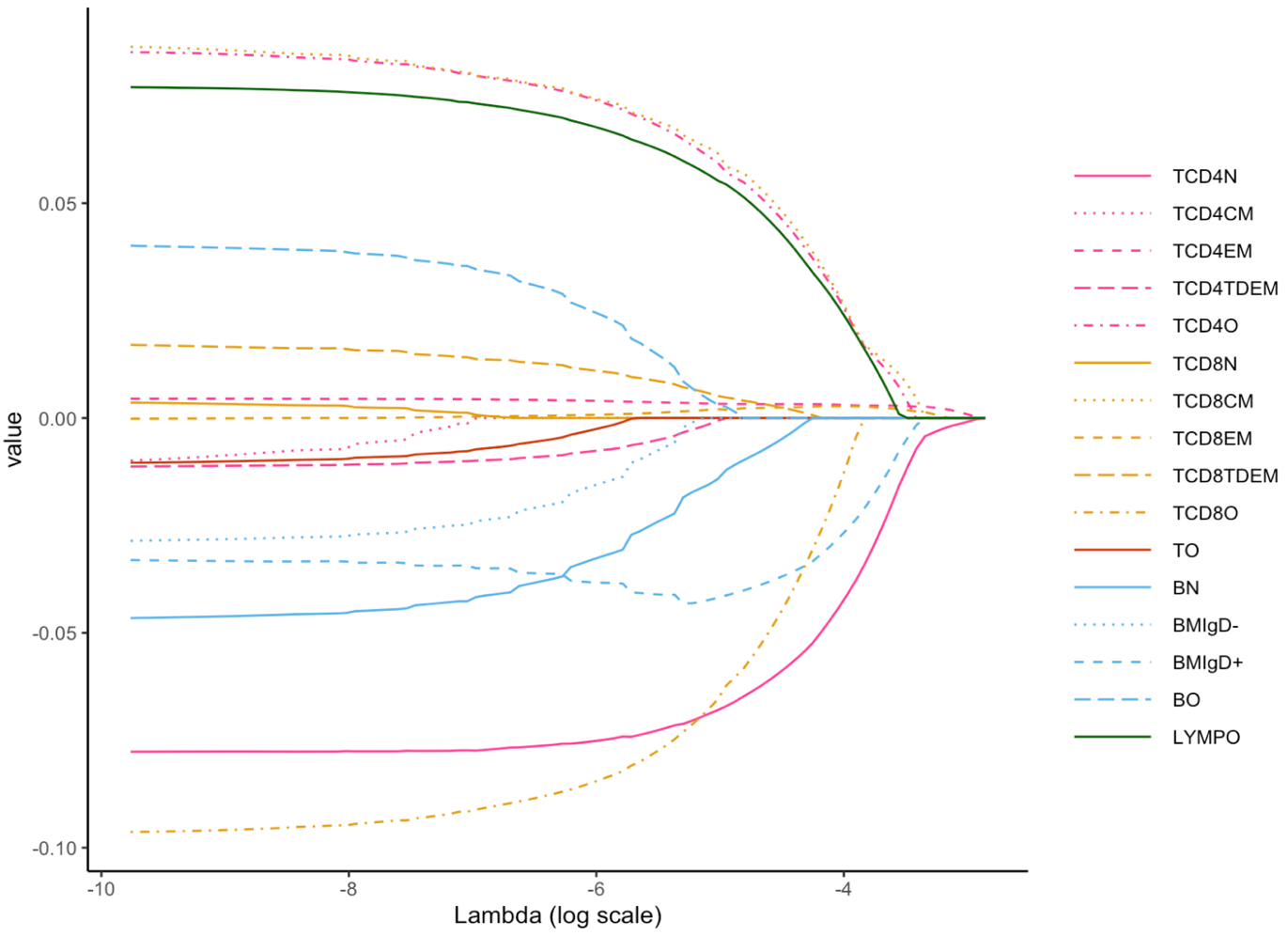
**

Footnote: The x-axis represents the log-transformed lambda values, and the y-axis indicates the estimated coefficient values. The plot illustrates how the coefficient for each cell type changes with the tuning parameter lambda. Larger lambdas impose greater penalization on non-zero coefficients, resulting in coefficients shrinking to zero as lambda increases.

**Supplementary Table 1. Demographic characteristics of individuals with and without missing data on T and B cells and their subsets**

|  | **With Missing Data** | | | **Without Missing Data** | | |
| --- | --- | --- | --- | --- | --- | --- |
|  | Median/Count | IQR/Percentage | | Median/Count | IQR/Percentage | |
| **Age (years)** | 68 | (61.0, 78.0) | | 67 | | (60.0, 76.0) |
| **Gender** |  | | |  | | |
| Men | 1225 | 40.4% | | 2841 | | 41.2% |
| Women | 1807 | 59.6% | | 4059 | | 58.8% |
| **Race** |  | | |  | | |
| Non-Hispanic White | 1993 | | 65.7% | 4386 | | 63.6% |
| Non-Hispanic Black | 551 | | 18.2% | 1196 | | 17.3% |
| Hispanic | 402 | | 13.3% | 1074 | | 15.6% |
| Other | 82 | | 2.7% | 231 | | 3.3% |
| Missing | 4 | | 0.1% | 13 | | 0.2% |
| **Education** |  | | |  | | |
| Below secondary | 253 | | 8.3% | 606 | | 8.8% |
| Lower secondary | 331 | | 10.9% | 746 | | 10.8% |
| Upper secondary | 928 | | 30.6% | 2056 | | 29.8% |
| Above upper secondary | 1508 | | 49.7% | 3456 | | 50.1% |
| Missing | 12 | | 0.4% | 36 | | 0.5% |
| **CMV** |  | | |  | | |
| Non-reactive | 838 | | 27.6% | 1949 | | 28.2% |
| Reactive or Borderline | 2140 | | 70.6% | 4920 | | 71.3% |
| Missing | 54 | | 1.8% | 31 | | 0.4% |

**Supplementary Table 2. Correlations among chronic disease index, self-reported health, and frailty level**

|  | **Chronic disease index** | **Self-reported health** | **Frailty level** |
| --- | --- | --- | --- |
| **Chronic disease index** |  | p<0.05 | p<0.05 |
| **Self-reported health** | 0.37 |  | p<0.05 |
| **Frailty level** | 0.60 | 0.54 |  |

Footnote: Kendall tau correlation coefficient was calculated for the ordinal categorical variables.

**Supplementary Table 3. Cumulative and additional variances of by each logratio among the 15 linearly independent logratios based on SRDA**

| **Logratios** | **Cumulative** | **Additional** |
| --- | --- | --- |
| TCD4N/TCD8TDEM | 0.265 | 0.265 |
| LYMPO/TCD4N | 0.442 | 0.177 |
| TCD4EM/TCD4O | 0.574 | 0.132 |
| LYMPO/TCD4CM | 0.659 | 0.085 |
| TCD4N/TCD4O | 0.727 | 0.068 |
| LYMPO/BN | 0.790 | 0.062 |
| TCD4TDEM/TCD8O | 0.834 | 0.044 |
| TCD4N/TCD8N | 0.873 | 0.039 |
| LYMPO/TCD8EM | 0.906 | 0.033 |
| TO/TCD4CM | 0.938 | 0.032 |
| LYMPO/TCD4TDEM | 0.962 | 0.023 |
| LYMPO/BMIgD- | 0.980 | 0.018 |
| BMIgD+/BO | 0.989 | 0.010 |
| TCD4N/TCD8CM | 0.997 | 0.007 |
| TCD4CM/BMIgD+ | 1.000 | 0.003 |
